# Left Superior Pulmonary Vein Rhythm Masquerading as Sinus Rhythm

**Published:** 2011-02-08

**Authors:** Rajiv Mahajan, Han S Lim, Dennis H Lau, Prashanthan Sanders

**Affiliations:** Cardiovascular Research Centre, Department of Cardiology, Royal Adelaide Hospital and the Discipline of Medicine, University of Adelaide, Adelaide, Australia

**Keywords:** Left Superior Pulmonary Vein Rhythm, Sinus Rhythm

A 55 year old male with a history of frequent symptomatic episodes of paroxysmal atrial fibrillation (AF) for four years was referred to us for catheter ablation. He had earlier tried flecainide and sotalol. However he was unresponsive to both of them. The echocardiogram was normal. Figure 1, left panel demonstrates his baseline electrocardiogram. The relatively slow rate and regularity of this rhythm was suggestive of sinus rhythm. However, notably the P wave morphology demonstrates positive P wave in V1, negative in aVL, isoelectric in lead I and notched and positive in inferior leads suggested that the rhythm was originating from the left atrium. The patient underwent catheter ablation during which mapping confirmed the origin of this slow rhythm (Figure 2, left panel) from the left superior pulmonary vein and that it competed with activity from the sinus node (Figure 2, right panel). Furthermore, fast tachycardia and atrial fibrillation was also noted to arise from this vein. Isolation of the left superior pulmonary vein eliminated all tachycardia and normalised the P wave on electrocardiogram (Figure 1, right panel). His procedure was completed with isolation of all four pulmonary veins by circumferential ablation.

The role of the pulmonary veins in triggering AF is well established [1]. Increasingly it is recognized that these structures may be responsible for atrial tachycardia and the maintenance of AF [2,3]. Boineau and colleagues have undertaken elegant experimental and clinical studies of sinus node activity and have demonstrated that such activity could arise from a variety of locations including the left atrium [4,5]. Yamane et al have previously reported a case of a pseudo-sinus rhythm arising from the pulmonary veins in a patient with AF [6]. In our case, we have observed a slow rhythm which competed with sinus node activity, triggers initiating AF and also bursts of rapid high frequency activity which could potentially maintain AF; all of which emanated from the left superior pulmonary veins. It provides further evidence of the spectrum of arrhythmias that can arise from the pulmonary veins in a patient with AF.

## Figures and Tables

**Figure 1:**
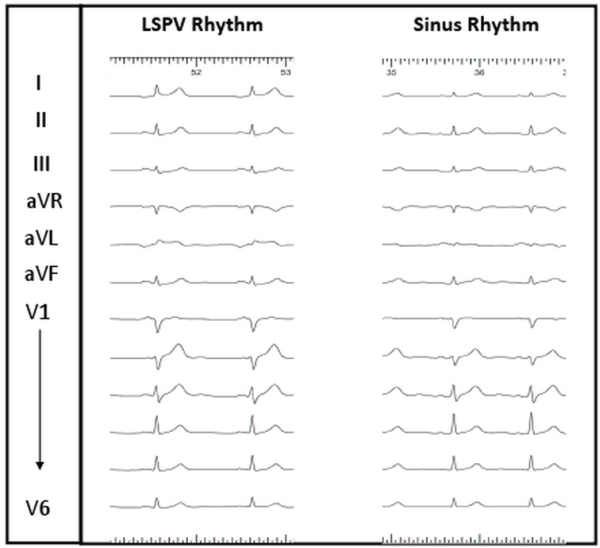
The left panel demonstrates the baseline rhythm at 60 beats per minute. The P wave is positive in lead V1, isoelectric in lead I, inverted in aVL and and positive and notched in inferior leads. This suggests that this rhythm is originating from the left atrium. The panel on the right demonstrates the ECG after isolation of the left superior pulmonary vein. The surface ECG in sinus rhythm demonstrates a P wave positive in I and aVL and biphasic in V1. LSPV - Left Superior Pulmonary Vein

**Figure 2:**
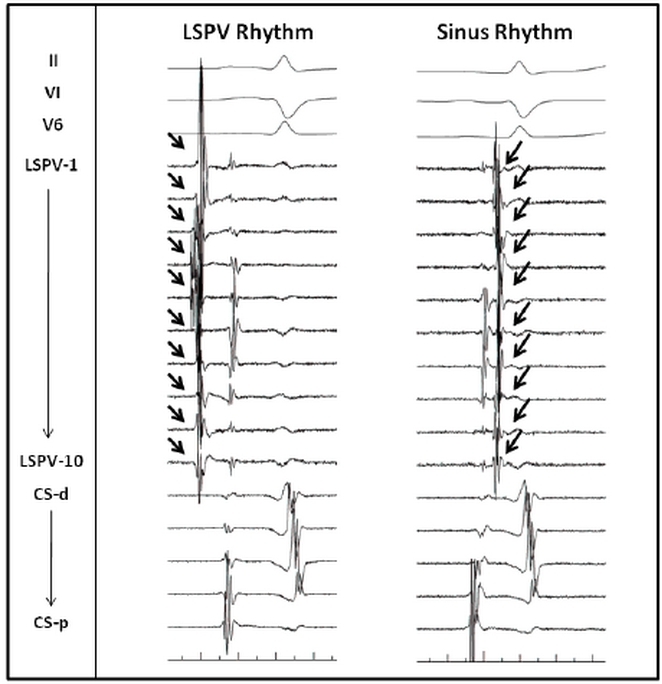
The intracardiac electrograms during the pseudosinus rhythm from the left superior pulmonary vein is demonstrated on the left. The Lasso catheter was placed at the left superior pulmonary vein. Note that the pulmonary vein potentials (arrows) precede the far field left atrial potentials. The right panel demonstrates the intracardiac electrograms during the sinus rhythm. The Lasso catheter was placed at the left superior pulmonary vein. The pulmonary vein potentials (arrows) follow the far field left atrial potentials.
